# Flash and grab: deep-diving southern elephant seals trigger anti-predator flashes in bioluminescent prey

**DOI:** 10.1242/jeb.222810

**Published:** 2020-05-19

**Authors:** Pauline Goulet, Christophe Guinet, Claudio Campagna, Julieta Campagna, Peter Lloyd Tyack, Mark Johnson

**Affiliations:** 1 Sea Mammal Research Unit, University of St Andrews, St Andrews, Fife KY16 8LB, UK; 2 Centre d'Etudes Biologiques de Chizé, CNRS, 79360 Villiers en Bois, France; 3 Wildlife Conservation Society, Argentina and Marine Programs, Amenábar 1595, 1426 Buenos Aires, Argentina; 4 Centro para el Estudio de Sistemas Marinos, CONICET, Boulevard Brown 2915, 9120 Puerto Madryn, Chubut, Argentina; 5 Department of Biology, Aarhus University, 8000 Aarhus, Denmark

**Keywords:** Anti-predator tactic, Bioluminescence, Biologging, *Mirounga leonina*, Foraging ecology, Predator–prey interactions

## Abstract

Bioluminescence, which occurs in approximately 80% of the world's mesopelagic fauna, can take the form of a low-intensity continuous glow (e.g. for counter-illumination or signalling) or fast repetitions of brighter anti-predatory flashes. The southern elephant seal (SES) is a major consumer of mesopelagic organisms, in particular the abundant myctophid fish, yet the fine-scale relationship between this predator's foraging behaviour and bioluminescent prey remains poorly understood. We hypothesised that brief, intense light emissions should be closely connected with prey strikes when the seal is targeting bioluminescent prey that reacts by emitting anti-predator flashes. To test this, we developed a biologging device containing a fast-sampling light sensor together with location and movement sensors to measure simultaneously anti-predator bioluminescent emissions and the predator's attack motions with a 20 ms resolution. Tags were deployed on female SES breeding at Kerguelen Islands and Península Valdés, Argentina. *In situ* light levels in combination with duration of prey capture attempts indicated that seals were targeting a variety of prey types. For some individuals, bioluminescent flashes occurred in a large proportion of prey strikes, with the timing of flashes closely connected with the predator's attack motion, suggestive of anti-predator emissions. Marked differences across individuals and location indicate that SES do exploit bioluminescent organisms but the proportion of these in the diet varies widely with location. The combination of wideband light and acceleration data provides new insight into where and when different prey types are encountered and how effectively they might be captured.

## Abstract (precis)

**Highlighted Article:** Description of a new measurement method and its successful application to deep-diving elephant seals, showing that these predators attempt to catch mesopelagic prey that react with bright anti-predator flashes.

## INTRODUCTION

The mesopelagic zone of the world's oceans is defined as receiving less than 1% of incident sunlight and the main source of light in this zone at night or in waters deeper than 500 m during the day is from bioluminescent organisms, which constitute an estimated 80% of all mesopelagic fauna (Haddock et al., 2010). Yet, much remains to be learnt about the ecological role of bioluminescence, especially in predator–prey interactions. Marine bioluminescence can take the form of a low-intensity continuous glow [e.g. for counter-illumination (Claes and Mallefet, 2008) or prey/mate attraction (Haddock et al., 2010)], or fast repetitions of higher intensity flashes aimed at distracting predators or exposing them to secondary predators (Hanley and Widder, 2017). Myctophids, a key constituent of the diurnally migrating deep scattering layer, dominate the mesopelagic fish assemblage, with an estimated biomass exceeding 11 gigatons (Irigoien and Klevjer, 2014). All myctophid species possess arrays of ventral and lateral photophores, which produce long-lasting glows used for counter-illumination, masking their silhouettes by replacing the downwelling light blocked by their bodies (Case et al., 1977). In addition, many species of myctophids possess patches of luminous tissues that can produce either single flashes with durations as brief as 40–80 ms or trains of flashes with repetition rates up to 30 Hz (Mensinger and Case, 1997). Such flashes are usually 1–2 orders of magnitude more intense than glows used for counter-illumination, in keeping with the goal of illuminating or temporarily dazzling dark-adapted predators (Barnes and Case, 1974).

The southern elephant seal (*Mirounga leonina*; hereafter, SES) is a major consumer of mesopelagic organisms (Hindell et al., 2003) including myctophids (Cherel et al., 2008). This apex predator inhabits sub-Antarctic islands during breeding and moulting seasons, but spends approximately 8 months of each year on trips to remote, deep-water foraging grounds (Guinet et al., 2014). Despite extensive studies of their movement and diving behaviour, relatively little is known about how SES find and choose prey in the deep ocean. On large spatial and temporal scales, SES are thought to rely on oceanographic features such as eddies and upwellings that aggregate mid-water prey (Bailleul et al., 2010; Campagna et al., 2006). However, on smaller scales, SES most likely use a combination of whiskers, audition and vision (relying on both residual downwelling light and bioluminescent light) to detect and catch individual prey (Levenson and Schusterman, 1999; Miersch et al., 2011). Foraging tactics may vary according to the type of prey being taken, and there is indirect evidence of prey switching (Jouma'a et al., 2017). Myctophids constitute an important part of the diet of female SES that breed on the Kerguelen Islands (Cherel et al., 2008) but the foraging depths of these seals at night-time (150–600 m; Vacquié-Garcia et al., 2015) do not always match those of the myctophids species found in the same area, most of which migrate at night to waters shallower than 200 m (Duhamel et al., 2000). This suggests that at night, SES may also target deeper myctophid species or other prey (Backus et al., 1968). However, collecting direct evidence of the prey types targeted by deep-diving marine predators as a function of time and space remains a technological challenge. Animal-borne cameras can potentially be used to identify nekton that are approached closely (McGovern et al., 2019; Naito et al., 2013; Thiebot et al., 2016), providing a definitive description of diet when image quality is good, but short battery lifetimes and the need for an external light source, with its attendant risk of disrupting predator and prey behaviour, limit their utility for long deployments on SES. A more energy efficient but perhaps less definitive approach could be to distinguish prey types on the basis of the bioluminescent signals that they emit, and this may have the added benefit of helping to interpret the sensory cues utilised by their seal predators.

The bioluminescent scenes encountered by deep-diving predators have yet to be reliably described, mainly because of the technical difficulty of detecting the extremely low light levels generated by normal bioluminescence in organisms (e.g. glow emissions of 10^8^–10^9^ photon s^−1^; Mensinger and Case, 1990). In contrast, anti-predator light emissions, although very brief, can be several orders of magnitude stronger and are most likely to be produced by prey that are close to the predator, potentially making these light levels more readily detected given the square-law attenuation of light intensity with distance. Light level measurements made by animal-borne tags on SES have demonstrated widely varying illumination at depth, with increased levels being generally linked with increased foraging activity and therefore probably related to anti-predator emissions (Campagna et al., 2001; Vacquié-Garcia et al., 2012, 2017). However, these studies used sensors with low sampling rates and long time constants that are optimised for measuring slowly varying light levels, and therefore probably under-represent short, bright anti-predator flashes. As a result, the dynamic, fine-scale relationship between SES hunting behaviour and bioluminescence remains unclear.

Here, we tested the hypothesis that brief but relatively intense light emissions should be closely connected with prey strikes when SES are targeting bioluminescent prey. The presence or absence of such emissions during prey captures should therefore provide information on prey type and capture tactics. To test this hypothesis, we used a biologging tag containing a sensitive fast-sampling light sensor together with high-resolution position and movement sensors to simultaneously measure prey bioluminescence and the seal's attack motions with 20 ms resolution. Tags were deployed on seals in two colonies (Kerguelen Islands and Península Valdés) to explore how encounters with bioluminescent prey vary in the contrasting foraging environments available to these animals.

## MATERIALS AND METHODS

### Data collection

Southern elephant seals, *Mirounga leonina* (Linnaeus 1758), were tagged in two locations. In October 2017 and 2018, three and two post-breeding female SES on the Kerguelen Islands (49°18′S, 70°32′E; KER) were equipped with a head-mounted DTAG-4 sound and movement tag (dimensions 97×55×33 mm, 200 g in air), a neck-mounted Argos tag (SPOT-293 Wildlife Computers, 72×54×24 mm, 119 g in air) and a back-mounted CTD-Fluorescence tag (SMRU-SRDL, 115×100×40 mm, 680 g in air). In October 2018, a further two individuals were tagged at Península Valdés, Argentina (PV) following the same protocol. Animals were anaesthetised while hauled out and tags were glued to the pelage using quick-setting Araldite adhesive with a low exothermal reaction. Total handling time of each animal was less than 1 h, and vital signs were monitored carefully throughout the procedure. See Jouma'a et al. (2017) for details of animal treatment. Tags were retrieved using the same anaesthetic procedure in December/January when the animals came back ashore to moult. Ethical approval was provided by the French Committee for Polar Environment and the University of St Andrews Animal Welfare and Ethics Committee.

A modified version of the DTAG-4 was developed for this study. This tag samples triaxial acceleration (200 Hz), triaxial magnetometer (50 Hz), depth (50 Hz), GPS (up to every minute) and sound (48 kHz). The tags were modified to include a light sensor sampled synchronously with the other sensors at 50 Hz. All sensors were sampled with 16-bit resolution. The light sensor comprised a photodiode (Hamamatsu S2387-1010R), operated in photoconductive mode, and a low-noise, linear preamplifier. To accommodate the wide dynamic range of light levels experienced by a deep-diving animal, the preamplifier gain was automatically varied by the tag software between four settings. The gain was lowered (or increased) by one setting if the light level was consistently above a high threshold (or below a low threshold) for 30 s, and the time of this change was logged by the software. Despite this gain control, transient light levels frequently exceeded the clipping level of the preamplifier and so the recorded light levels were kept in relative units of 0–1 rather than attempting to estimate the photon density at the photodiode face. The photodiode was mounted to the front part of the tag facing forward, facilitating detection of bioluminescent sources ahead of the animal. The resulting tag was powered by three AA lithium thionyl chloride batteries and had 64 GB of memory, allowing a continuous recording of audio, with loss-less compression, GPS, movement and light data for 30 days. The tag electronics, sensors and battery were cast in clear epoxy to create a single compact pressure-tolerant unit.

### Data analysis

The tags were configured to start operating soon after animals left the haul-out and so began recording while the seals were still in shallow continental shelf waters for an interval of 3–24 days. Although seals may forage opportunistically as they head towards deeper water, the large majority of foraging in the 2–3 month post-breeding trip occurs in deeper waters; therefore, the analysis focused on dives taking place beyond the shelf break, i.e. in waters deeper than 1000 m (Guinet et al., 2014). All data analyses were conducted in Matlab (version R2018b) using custom-written code and functions from www.animaltags.org.

### Prey capture attempts

Prey capture attempts (PCAs) were detected in the acceleration data using the root mean square (RMS) of the norm jerk, i.e. the RMS of the vector magnitude of the rate of change in the 3-axis acceleration (Ydesen et al., 2014). The RMS jerk was computed from the full bandwidth (200 Hz sampling rate) acceleration data using an averaging time of 40 ms and an overlap between averages of 20 ms to give an output sampling rate of 50 Hz. A delay-corrected symmetric finite impulse response (FIR) filter was used for the averaging. RMS jerk histograms for each animal showed a bimodal distribution with a minimum value of about 350 m s^−3^ separating the low and high jerk modes (Fig. S1). The low jerk mode was associated with swimming and resting behaviours while the higher jerks occurred as brief transients during deep dives and so presumably indicate PCAs. This value was therefore used as a threshold to detect PCAs on all animals. PCAs occurring shallower than 20 m were excluded from the analysis to eliminate jerk transients due to surfacing. The start and end times of each PCA were defined as the first and last times that the RMS jerk exceeded 350 m s^−3^. Log-survivor curves of the time interval between jerk transients showed a change of slope at 5 s and this was used as the minimum interval between two PCAs for them to be counted separately. For each PCA, its duration, peak RMS jerk value, and time and depth of occurrence were recorded.

### Light data processing

Sections of overloaded light data or light not recorded at the maximum preamplifier gain (i.e. corresponding to the highest sensitivity) were excluded from the analysis as these necessarily occurred when the ambient light level was high and anti-predatory flashes of bioluminescence would therefore be less likely to be detected. To remove the slowly varying direct current offset of the preamplifier, and aid detection of short transient flashes, a delay-corrected FIR high-pass filter (cut-off frequency 0.5 Hz) was applied to the raw light level data.

The GPS in the tag uses an infrared (940 nm) reflectometer to detect when the antenna is out of the water and this device produces a short (approximately 100 µs) flash of light every 101 ms, which is picked up by the bioluminescence sensor resulting in a stereotyped spike every five samples. In the tags deployed in 2018, the infrared LED was disabled at depths below 50 m to avoid this interference. For the tags deployed in 2017, an additional processing step was required to remove the interference: the light data were divided into sequences of 101 samples (the repetition interval of the resulting interference pattern at 50 samples s^−1^) and the median values of samples across multiple sequences were subtracted from the original data. This method was highly effective at removing the interference because of the very stable coupling between the infrared LED and the photodiode.

### Flash detection

Brief flashes of light were recorded with varying frequency by the light sensors throughout the deployments. As SES are thought to forage within the highly bioluminescent deep scattering layer (DSL), these flashes could arise spontaneously from nearby bioluminescent organisms or may be provoked by the seal's swimming motions. However, some flashes occurred in close proximity to PCAs and so could indicate that bioluminescent prey were targeted. To identify how many flashes occurred during PCAs purely by happenstance, we performed a bootstrap test on intervals of similar length to PCAs during which seals were searching for, but not actively capturing, prey. Intervals of 10 s duration, approximately equal to the upper quartile of PCA duration, beginning 20 s before the start time of each PCA, were extracted from the light data. Any intervals falling during another PCA or recorded at low preamplifier gain were removed, resulting in a set of light vectors that should be unrelated to prey encounters (given a forward speed for SES of 1–1.5 m s^−1^, targeted prey would be >10 m away from the seal 10 s before a PCA, which well exceeds the likely detection distance of the light sensor). The peak light intensity value in each such vector was found and the 98th percentile of these for each animal was taken as an individual-specific flash detection threshold with a constant 2% false alarm rate. This threshold was then applied to the full light level recording at high gain to detect flashes. As for PCAs, flashes separated by more than 5 s were considered to be separate. The start and end times of each flash were defined as the first and last times the light level exceeded the detection threshold. This included the recovery time of the light sensor, which was 0.04 s for low flash intensities and 0.2 s when the sensor was overloaded. Flashes recorded at depths shallower than 20 m were excluded from the analysis in order to match the constraints on PCA detections near the surface and avoid false detections. As flashes frequently overloaded the sensor for one of the individuals, we focused analysis on the timing and duration of the flashes rather than their intensity.

### Flash–PCA associations

Flashes that started within ±5 s of a PCA start time were identified as potential flash–PCA associations. For each such event, the flash duration, PCA duration and depth of occurrence were recorded. We expect about 2% of PCAs to register a flash by chance as a result of the choice of flash detection threshold, i.e. in *n* independent PCA events, we expect 0.02*n* false alarms. Indeed, if flash occurrence is unrelated to PCAs, the number of observed flash–PCA associations in *n* PCAs should follow a binomial distribution (with parameters *p*=0.02 and *n*) giving the probability of observing more than *k* flash–PCA associations as:(1)
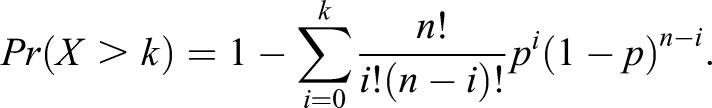
To identify individuals with foraging modes that are linked with bioluminescence, we calculated the lowest value of *k* that gave *Pr*(*X > k*)<0.01 (i.e. the probability of observing *k* or more flash–PCA associations in *n* PCAs by chance is less than 1%).

### Solar and oceanographic data

Solar angles were calculated for each day of the deployment using the first GPS position of the day, and a three-level day/night factor was associated with each PCA, flash and flash–PCA association, calculated as follows: day (sun angle >0 deg), twilight (sun angle 0–12 deg below horizon), night (sun angle >12 deg below horizon) (Blanchet et al., 2013). Twilight data (which comprise 12.5% of the tag recordings) were included in total PCA counts but were not examined for flashes and flash–PCA associations because of the rapidly changing dive depths and surface illumination at these times.

Sea surface temperature (SST) averaged over the study periods was obtained from AQUA-MODIS at 0.1 deg spatial resolution. GPS and raster data were processed using QGIS v3.6.3.

## RESULTS

### Field deployments

**Fig. 1.**
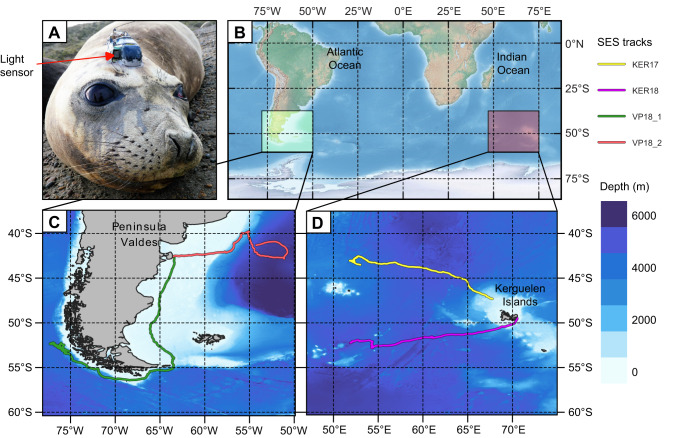
**GPS tracks obtained from the field deployments.** (A) A DTAG with light sensor deployed on a female southern elephant seal (SES). (B–D) Tagging location (B) and GPS tracks of the four individuals tagged in 2017 and 2018 at Peninsula Valdes, Argentina (PV; C) and the Kerguelen Islands (KER; D).

Seven DTAGs with light sensors were deployed in October 2017 and 2018, only four of which were recovered as three seals moulted on inaccessible islands in the Kerguelen archipelago (Fig. 1, Table 1). The tags recorded continuous high-resolution movement, location, audio and light data for 29–54 days. Excluding time spent in shallow water, a total of 102 days of data were available for analysis. The two individuals from KER spent most of their time in deep waters west of the Kerguelen shelf. One seal tagged at PV (PV18_1) headed east and crossed the continental shelf after 6 days to forage in deep, offshore waters. In contrast, the other individual (PV18_2) travelled south for 24 days over the shallow Patagonia shelf before reaching the continental slope off the Chilean coast, where it foraged in deeper waters for the remainder of the tag recording.

**Table 1.**
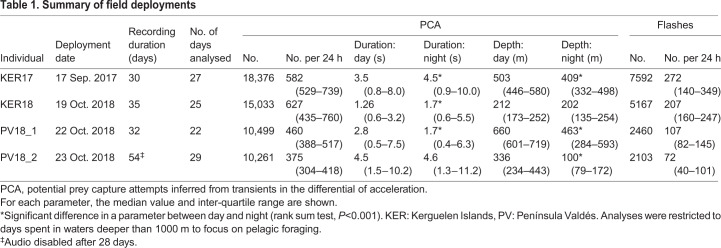
**Summary of field deployments**

### PCAs

The number of potential PCAs, as inferred from jerk transients, averaged 584 day^−1^ for the KER seals and 398 day^−1^ for the PV seals (Table 1). PCAs at night were consistently shallower than those during the day for all animals except KER18 (individual rank sum tests, *P*<0.001). For both KER individuals, PCAs were significantly longer at night (individual rank sum tests, *P*<0.001), whereas for the PV animals, PCA durations either did not change significantly between day and night (PV18_2) or were longer during the day (PV18_1).

### Bioluminescent flashes

More than 2000 bioluminescent flashes were detected for each of the four animals, with flashes occurring in a large number of dives that were deep enough for the light sensor to attain its highest gain setting (Table 2). The number of flashes per day varied by individual from 72±42 (median±interquartile range, IQR) to 271±213, with relative flash intensities averaging 0.9 and frequently overloading for KER17, and 0.3 for the three individuals tagged in 2018.

**Table 2.**
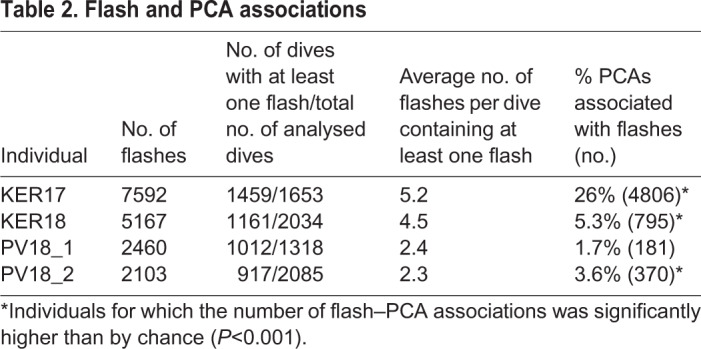
**Flash and PCA associations**

Notably higher flash rates and intensities were recorded by the tag on KER17, but this is unlikely to be due to a difference in sensitivity of this tag as the same unit was reused on KER18. We examined whether this strong difference could be related to the different software used in the two years; specifically, the tag in 2017 used an infrared LED to detect immersion and this produced stereotyped spikes in the light data which were removed in post-processing. We checked whether flashes remaining after this post-processing step were correlated with the infrared emissions by removing the light values recorded when the LED fired, as well as the samples immediately before and after to allow for small timing errors. This should remove the majority of flashes if they are caused by the infrared emissions. The number of flashes detected in the sub-sampled data remained about the same, confirming that the large majority of the flashes detected for KER17 were likely to be real bioluminescent events.

### Flash and PCA associations

**Fig. 2.**
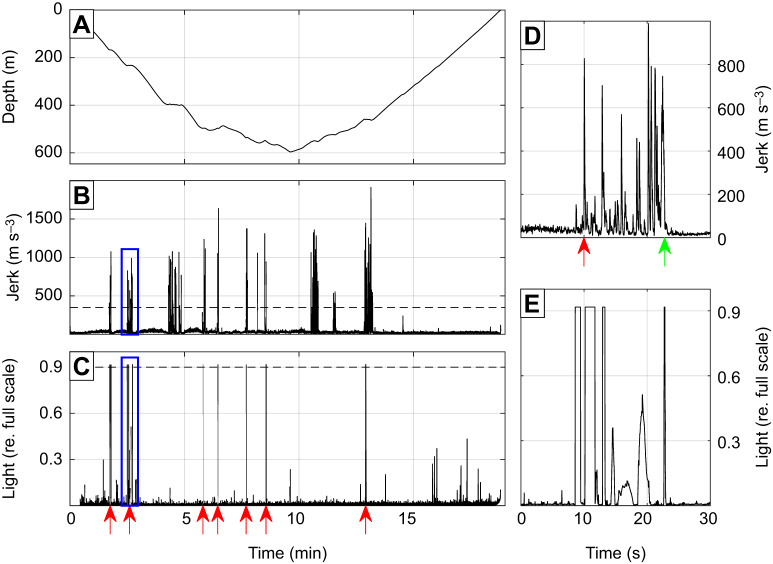
**Depth, jerk and light data during a 20 min foraging dive (individual KER17).** (A) Depth data. (B) Jerk data. Transients above the threshold at 350 m s^−3^ (dashed line) separated by at least 5 s were counted as individual prey capture attempts (PCAs). (C) Light data over the same interval. Transients above the high threshold (dashed line) separated by >5 s were counted as bioluminescent flashes. Flashes occurring within 5 s of a PCA start time are indicated by red arrows. (D,E) Detailed views of jerk (D) and light (E) data during a flash–PCA association showing close synchronisation between the two signals (the timing of the insets is indicated by the blue rectangles in B and C). Arrows indicate the PCA start time (red) and end time (green).

**Fig. 3.**
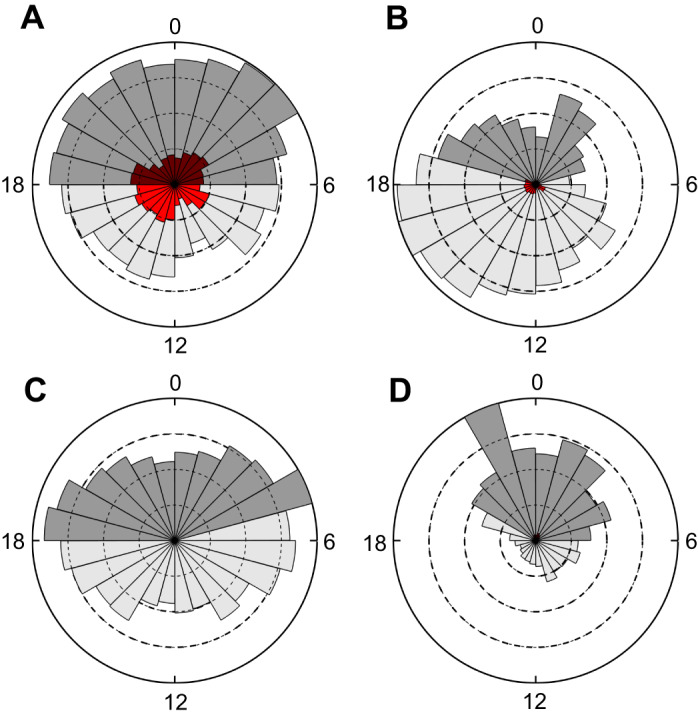
**Number of PCAs per hour of the day (local time) summed across the entire trip for each of the tagged individuals and normalised to the highest number of PCAs per hour for each animal.** The proportion of PCAs with bioluminescent flashes relative to the total number of PCAs for each time slot is indicated by a red colour and darker shades indicate nocturnal captures. (A) KER17. (B) KER18. (C) PV18_1. (D) PV18_2.

Bioluminescent flashes were detected in close connection with PCAs (i.e. within 5 s of PCA start time) in all four datasets (Fig. 2). However, the occurrence rate of these compared with similar length intervals without PCAs was strongly significant (*P*<0.01) in only 3 out of 4 individuals (Table 2). Within these three seals, the number of flashes associated with PCAs varied widely between individuals but only comprised >5% of all PCAs, and therefore a sizeable fraction of the diet, in the KER animals (Fig. 3). As a result, the relationship between flashes and PCAs was investigated further in these two animals only. The median duration of individual flashes associated with PCAs was 0.46 s (IQR 0.22–0.86 s) for KER17 and 0.22 s (IQR 0.08–0.44 s) for KER18.

### Prey switching

**Fig. 4.**
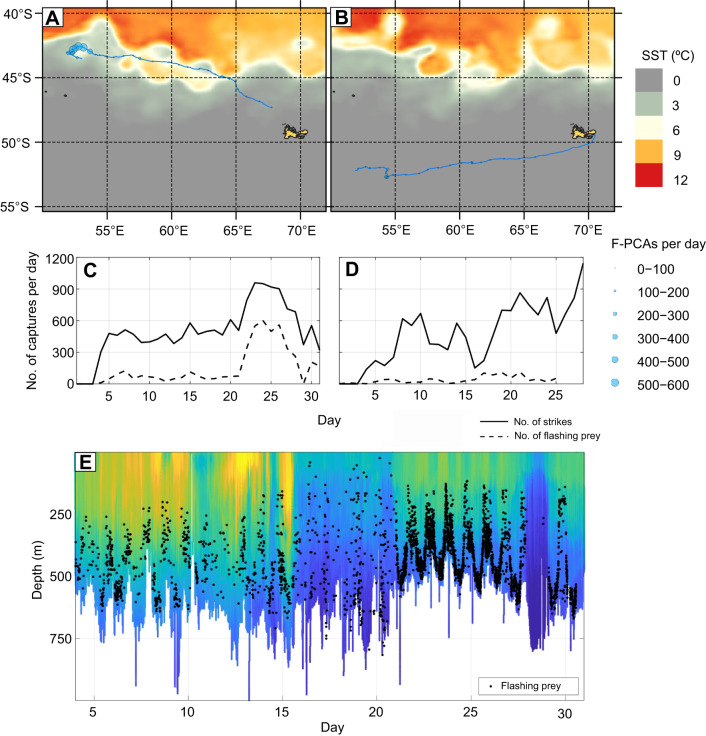
**GPS tracks, and number and depth of** **PCAs** **associated with a flash.** (A,B) GPS tracks of the two SES tagged on Kerguelen Island in 2017 and 2018 (A: KER17; B: KER18) along with sea surface temperature (SST). The dot size indicates the number of flashing (F-)PCAs per day. (C,D) Number of PCAs per day with and without an associated flash for each animal (C: KER17; D: KER18). (E) Time and depth of individual PCAs associated with a flash overlaid on the high-resolution (1 Hz) temperature profile recorded by the CTD tag attached to the back of KER17.

**Fig. 5.**
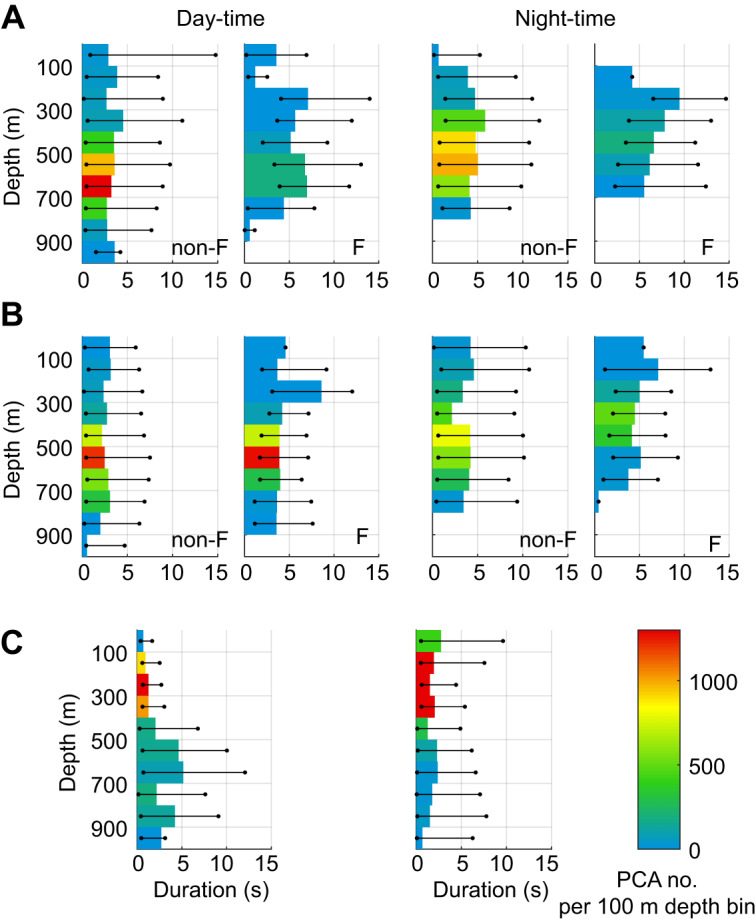
**Duration and number of PCAs in 100 m depth bins occurring during the day-time (left) and night-time (right).** (A) PCAs performed by KER17 for days 3–18 for non-flashing prey (non-F) and flashing prey (F). (B) PCAs performed by KER17 for days 19–31 (corresponding to time spent in a warm water gyre) for non-flashing prey (non-F) and flashing prey (F). (C) All PCAs performed by KER18 for days 4–28. The bars indicate the median duration of the PCA occurring in each depth bin, along with the 25th and 75th percentiles in PCA duration represented by the horizontal black lines. The colour scale indicates the number of PCAs occurring in a given 100 m depth bin.

For both KER animals, the occurrence and depth of flash–PCA associations varied widely throughout the trip as well as with time of day. In particular, for KER17 a dramatic increase in bioluminescent events and flash–PCA associations occurred after day 18 (Fig. 4). For the subsequent 12 days, a daily average of 41% (maximum 57%) of PCAs were associated with flashes compared with an average of 12% (maximum 21%) in the preceding 15 days (the first three of which were spent in shallow water). This sudden change coincided with the seal foraging in an eddy of well-mixed, relatively warm water intruding south of the sub-Antarctic Front (Fig. 4). PCAs were consistently shorter in this mixed water area (median value for flashing prey 4.0 s, non-flashing prey 3.0 s) than in the first part of the trip (median value for flashing prey 6.8 s versus non-flashing prey: 4.1 s; rank sum *P*<0.001) (Fig. 5).

**Fig. 6.**
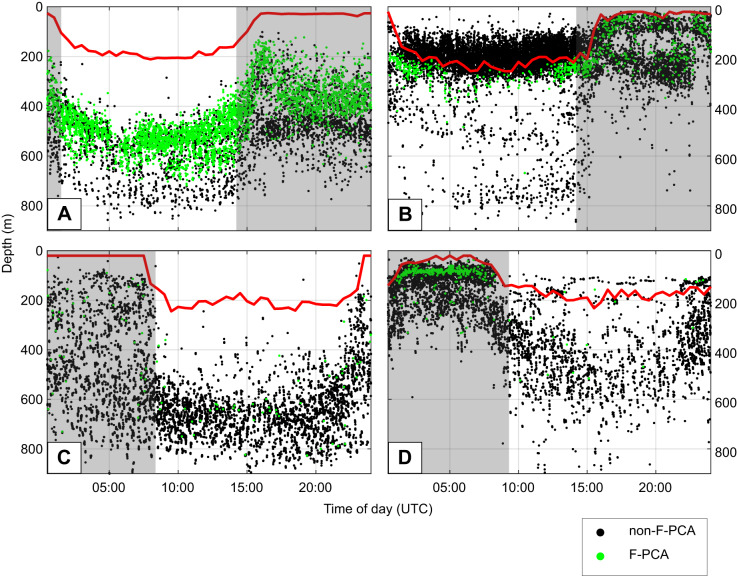
**Depth of occurrence of** **PCAs** **with and without an associated bioluminescent flash.** Each dot represents a PCA, with (green) or without (black) a flash. The grey background indicates night-time. Light level data were only processed when the light sensor was at its highest sensitivity, which depended on the ambient light level. The average depth at which the sensor switched to high sensitivity is shown by the red line. (A) KER17. (B) KER18. (C) PV18_1. (D) PV18_2.

Judging by the presence or absence of bioluminescent flashes, KER17 targeted two distinct layers of prey when foraging in the warm water eddy (Figs 5 and 6A): during the day, 87% of all PCAs occurred in a prey layer located at 400–600 m depth, half of which were associated with a flash, while a deeper layer from about 650 to 800 m depth contained the remaining day-time PCAs with less than 10% being flashing prey. At night, two prey layers were also encountered but at significantly shallower depths (rank sum test on dive depths, *P*<0.001), yet with a similar arrangement of a shallow layer (approximately 200–450 m) containing 75% of the night-time PCAs, 45% of which were associated with flashes, and a deeper layer (approximately 450–600 m) containing <5% flashing prey. This depth stratification was not observed during the first part of the trip.

KER18 also appeared to target two distinct prey layers during the day (Figs 5 and 6B): a small proportion of prey were found over a broad depth range (approximately 400–800 m) in which PCAs with flashes were absent. In contrast, 88% of the day-time PCAs occurred in a shallow layer from 100 to 320 m, 4% of which were associated with a bioluminescent flash. The PCA durations associated with these prey layers were distinct with a median duration of 3.5 s for the deep layer and 1.2 s for the shallow layer (rank-sum *P*<0.001). At night, two shallow patches were identifiable: one near the surface and a deeper one located from 200 to 300 m (Fig. 6B). These two layers contained the same proportion of flashing prey (7%). No significant difference in PCA duration was found between these layers or between flashing and non-flashing prey at night.

PV18_2 had a small but significant number of PCAs associated with flashes and some evidence of prey switching was found for this individual (Fig. 6C). This animal targeted a single prey layer during the day but exhibited a bimodal foraging behaviour at night: 60% of the night-time prey were taken in a layer shallower than 120 m (median PCA duration 1.1 s, IQR 5.8 s) while the remaining prey were taken in a broader deeper layer (median PCA duration 4.2 s, IQR 10.4 s). The small number of flashing prey encountered by this animal occurred in the shallow night-time layer.

In comparison, PV18_1 did not appear to find prey in discrete layers (Fig. 6D). This animal foraged over a 400–900 m depth range during the day and in an even broader range (100–900 m) at night.

### Flash–PCA timing

**Fig. 7.**
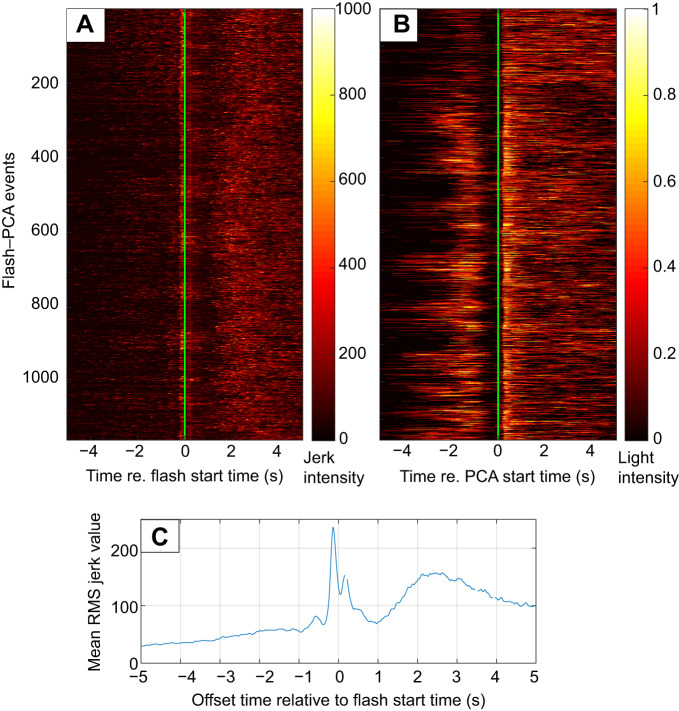
**Temporal relationship between jerk and light data for KER17.** Each flash–PCA event is represented as a horizontal coloured line. (A) Jerk data (10 s long segments) are vertically stacked and aligned with the flash start time (time 0, indicated by a green line). (B) Light data (10 s long segments) are vertically stacked and aligned with the PCA start time (time 0, indicated by a green line). (C) Mean RMS jerk value averaged over all flash–PCA events (*n*=4674) and synchronised to flash start time (time 0 s).

Flashes were counted as being associated with a PCA if they occurred within 5 s of the PCA start time. However, for both KER seals, the timing between flash and PCA was typically much closer than this. The start time offsets between flash and PCA for KER18 had a narrow (s.d.=2 s) unimodal distribution centred on zero, indicating a tight synchronisation between flash emission and start of the prey capture but with flashes starting equally often shortly before or after the PCA. The flash–PCA relationship was somewhat different for KER17: the flashes for this animal appeared to occur consistently before the PCA start. However, plotting sections of the jerk waveform in each PCA synchronised to the flash start time revealed a consistent brief and relatively small jerk occurring about 0.2 s before the flash start time (Fig. 7A). This jerk transient with a mean RMS level of 250 m s^−3^ was below the threshold used to detect the PCA start time but still clearly above the jerk level in regular swimming (Fig. 7C).

## DISCUSSION

Studying where marine predators find prey and what type of prey they attempt to capture is a challenging problem in biologging, prompting the development of novel animal-attached instruments (Davis et al., 1999; Goulet et al., 2019). Here, we examined whether data from high-resolution light and motion sensors can shed light on the prey targeted by deep-diving predators that are known to eat bioluminescent prey. Specifically, we sought to test the hypothesis that anti-predator flashes from bioluminescent prey would be closely associated with the predator's capture attempts. Detection of light levels during brief feeding encounters is complicated by the generally low intensity of bioluminescent events coupled with wide variation in ambient light conditions experienced by deep-diving predators, and by the high temporal resolution needed to acquire brief flashes and the associated reaction of the predator. To overcome these issues, we modified a biologging device to simultaneously record acceleration and light data with high sampling rate. We applied this device to SES with the goal of using the presence and absence of flashes during prey capture attempts to infer when these Southern Ocean predators were accessing different classes of prey and whether different tactics and effort were required to capture them.

All four of the tagged SES encountered bioluminescent prey to a varying extent throughout their foraging trips, perhaps reflecting both individual tactics and geographical differences in prey type. Two SES tagged on the Kerguelen Islands, which foraged in oceanic waters, encountered bioluminescent prey frequently and displayed stereotyped capture behaviour in association with this bioluminescent resource. These individuals switched between prey types, suggesting a continual adjustment of foraging tactics to match changes in their biotic environment. In comparison, both SES tagged in Península Valdés encountered fewer bioluminescent prey and displayed limited evidence for prey switching. Below, we discuss first the methodology used to detect foraging and bioluminescence with the new biologging tag and then consider what the results may indicate about prey availability and foraging tactics of SES.

### PCAs

The use of high sampling rate head-mounted triaxial accelerometers in this study made detection of feeding events straightforward. Applying the RMS jerk method proposed by Ydesen et al. (2014) to the 200 Hz accelerometer data gave clear discrete peaks during foraging dives that were readily distinguished from the lower jerk levels generated by swimming movements. The resulting PCA counts of around 550 day^−1^ for the KER animals are similar to prior studies from the same location that used lower bandwidth acceleration and a more complex detector (Richard et al., 2016). Thus, feeding rates inferred from acceleration transients seem to be robust, which is consistent with a strong, unambiguous head acceleration when elephant seals strike at prey.

PCA duration varied significantly between the two study sites and within individuals, both spatially and as a function of day/night. Following Ydesen et al. (2014), we suggest that PCA duration, essentially a measure of time investment in a capture, can help differentiate prey type or the agility of prey: assuming that a single prey is targeted within each PCA, a longer PCA could indicate a more alert and agile prey (requiring a longer chase) or a larger prey (associated with a longer handling time). Combining this with additional data on the prey such as its depth and bioluminescent emissions may help further distinguish prey types.

### Bioluminescent events

Brief distinctive transients consistent with bioluminescent flashes were recorded frequently by the tags when surface light levels were sufficiently attenuated. An average of 66% of analysed dives contained at least one such bioluminescent event (Table 2). However, only 12% of these flashing organisms were associated with a prey capture attempt in the PV animals, versus 40% for the KER animals, indicating that a large proportion of flashing organisms encountered by the PV animals were not targeted. For the KER animals, dives containing bioluminescent events contained an average of 5 flashes per dive. This exceeds the average of 1.6 flashes per dive found by Vacquié-Garcia et al. (2017) for KER seals but these values cannot be reliably compared because of the very different light sensors and analysis protocol. In particular, the present study sampled light levels from a photodiode at 50 Hz, versus 0.5 or 1 Hz for Vacquié-Garcia et al. (2012, 2017). The sensor used by Vacquié-Garcia et al. (2012, 2017) is most responsive to flashes longer than 1 s (Fig. S2) which account for only 27% of the flashes detected in the present study. Given this reduced bandwidth, the 1.6 flashes per dive reported by Vacquié-Garcia et al. (2017) is commensurate with our result (i.e. 5×0.27=1.4 flashes per dive predicted by our study). The frequent occurrence of much shorter flashes is consistent with measurements of anti-predator bioluminescent emissions in myctophids, which can be as brief as 40 ms (Mensinger and Case, 1990). In addition, the high dynamic range linear photodiode preamplifier in our tag enabled flash detections from 200 m depth during the day and from near the surface at night (Fig. 6). In comparison, the sensor in the Wildlife Computers Mk9/Mk10 tags used by Vacquié-Garcia et al. (2012, 2017) is primarily intended for ambient light level measurement for geolocation and employs a logarithmic transformation of the photodiode current to accommodate widely varying light levels, resulting in a sensitivity that depends on the background light level (Fig. S2). Perhaps for this reason, Vacquié-Garcia et al. (2012, 2017) were only able to report bioluminescent emissions below 550 m during day-time and 250 m at night. Thus, we conclude that a high sampling rate and dynamic range are critical in a light sensor to capture brief bioluminescent emissions over a wide depth range.

KER17 encountered considerably higher intensities and a higher overall rate of bioluminescent flashes than the other animals. We eliminated the possibility that the high flash rate was due to interference from an infrared LED immersion sensor which was disabled below 50 m in the tags deployed in 2018. We therefore conclude that the high rate and intensity of flashes recorded in 2017 indicate that this individual encountered a greater proportion of bioluminescent organisms with strong anti-predator emissions. This animal may also have a hunting tactic that enabled it to approach more closely before triggering anti-predator flashes, leading to stronger light levels impinging on the sensor.

### Association between flash and PCA

To test the hypothesis that anti-predator flashes should be closely connected with prey strikes, we looked for light transients that occurred within 5 s of each PCA start time, corresponding to a distance of <5–10 m assuming a forward speed of 1–2 m s^−1^. PCAs with a closely associated light flash occurred far more often than chance and represented more than 5% of the total PCAs in the seals from the Kerguelen Islands but not Península Valdés. Individual flashes had durations of 0.2–0.5 s, well under the 2 s duration suggested to discriminate between flash and glow bioluminescence (Mensinger and Case, 1997). This lends strong support to the notion that these are predator-deterrent light emissions; such emissions are 10–100 times brighter than bioluminescence used for signalling and counter-illumination and are emitted in short pulses (Mensinger and Case, 1990). Moreover, prey are likely to be close to the mouth at the start of the PCA, maximising the light level that arrives at the tag. In comparison, light levels arriving at the tag from glowing organisms near the seal will be much lower and will vary more slowly, making them difficult to distinguish from the background light level or the photodiode dark current. Given the sensitive eyesight of SES, these lower level emissions, serving for instance as a lure or for intra-specific signalling (Haddock et al., 2010), could nonetheless be used as a cue by the seals, enabling them to locate prey at a greater distance than that over which the tag can detect light.

Some 70% and 90% of PCAs performed by KER17 and KER18, respectively, were not associated with a flash and may have targeted non-bioluminescent prey. However, our light sensor is likely to be 1–2 orders of magnitude less sensitive than an elephant seal eye and so a proportion of these prey may have been bioluminescent, emitting a flash either too weak or too far away to be detected by the light sensor but which was nonetheless detected by the seal. The co-occurrence of both flashing and non-flashing PCAs in some shallow foraging dives by the KER seals could therefore indicate a mix of prey, or that there are organisms with different behavioural–ecophysiological status within a single prey type or conceivably that the seal is able to strike at, and capture, some prey without eliciting a response. Moreover, many of the day-time PCAs of KER18 occurred close to the depth at which the gain of the light sensor was adjusted down to avoid overloading from ambient light and were therefore excluded from the analysis. The proportion of bioluminescent prey for this individual may therefore be underestimated.

Despite a large number of flashes encountered by the PV animals, the proportion of PCAs associated with flashes was less than 4%. Such a rare occurrence of flash–PCA associations in the PV animals indicates that they are mainly pursuing prey that do not produce bioluminescence as a predator defence. This is consistent with the predominance of mid-water squid in the diet of female SES off the Península Valdés continental shelf (Slip, 1995), many of which are thought to be non-bioluminescent (Rodhouse et al., 1992). The relatively long PCA durations for these seals also suggests that they were targeting larger or harder to catch prey, which were probably more dispersed, as hinted at by the lower PCA rate.

The frequent occurrence of flash–PCA associations for KER17 allowed us to investigate more closely the interaction between this individual and flashing prey. About half of these anti-predatory flashes occurred synchronously with the start of the PCA, suggesting an anti-predatory light emission in response to imminent predation. The other half of flashes occurred about 2 s before the start of the PCA. However, these flashes consistently occurred shortly after a brief, lower intensity jerk peak representing a more subtle movement detected by the accelerometer, e.g. due to a small movement of the head or muscle contractions that expand the whiskers as the seal approaches its prey (McGovern et al., 2019; Naito et al., 2013). The resulting water movement could be mechanically sensed by the prey, inducing it to emit a defence flash (Barnes and Case, 1974).

Although overall foraging success cannot be inferred yet from accelerometer data, the duration of the PCAs may indicate how effective the flashes are in abating SES predation. The longer duration of PCAs with flashes, compared with those without, implies that flashing prey are more difficult to capture, requiring an extended chase, or involve more handling time. Multiple flashes were frequently recorded throughout the whole capture, indicating that these prolonged PCAs are more likely to be due to an increased chase time. This suggests that either bioluminescent prey are more vigilant and active or that flashing is somewhat effective as a predator-abatement tactic against SES (e.g. by dazzling dark-adjusted eyes), or a combination of the two.

### Implications for prey selection

Both KER individuals consistently targeted a group of deeper, non-flashing prey during the day, representing 10–30% of the day-time PCAs. These PCAs were consistently long, implying greater difficulty in capturing prey. We propose that these prey could be predators of the DSL with an energy content rewarding enough to compensate for the greater time and energy expenditure associated with these captures. However, 90% of KER18 day-time PCAs occurred in a shallow prey layer, located between 100 and 300 m depth and lasted less than 2 s, suggesting small or lethargic prey such as myctophids in a low-activity resting mode (Cherel et al., 2008). Although KER17 generally targeted deeper prey than KER18, the two tactics yielded comparable overall prey encounter rates that were significantly greater than those for the animals tagged in PV, probably reflecting the occurrence of different prey in these very different ecosystems.

The availability of bioluminescent prey varies widely by location as illustrated by KER17. This seal travelled north-west from the Kerguelen Islands and spent the first 18 days of its trip in stratified, relatively cold waters with an average of 50 flashing PCAs per day. During the following 12 days, however, KER17 entered a cell of well-mixed, warmer water (Fig. 4), south of the sub-Antarctic front, where it encountered a larger number of flashing prey (300 PCAs with flashes per day), associated with shorter capture durations, which may reflect either a change in the physiological state of the prey or, more likely, a switch in prey type. This latter hypothesis seems likely as eddies are known to host different ecological communities compared with the adjacent environment (Strass et al., 2002).

SES range widely during their long foraging trips, taking very different routes even when starting from the same haul-out location (Guinet et al., 2014). Thus, individual animals encounter diverse prey resources, making it fraught to predict overall foraging behaviour from a small dataset. As in other studies, the SES tagged in this study encountered extensive prey resources arrayed in vertical layers that show predictable diel movements but less predictable horizontal structure. Our results demonstrate that SES can exploit large quantities of bioluminescent prey but the reliance on these varies widely, as shown by the contrast between the two study locations. The combination of wideband light and acceleration sensors in a tag therefore provides new insight into where and when different prey types are encountered, and how effectively they might be captured, meriting their use in more extensive studies of SES and other deep-diving apex predators.

## Supplementary Material

Supplementary information
