# Supplementary material for: Flash and grab: deep-diving southern elephant seals trigger anti-predator flashes in bioluminescent prey
Source: J Exp Biol. 2020 May 19;223(10):jeb222810. doi: 10.1242/jeb.222810 (PMC7322542; doi:10.1242/jeb.222810)
Supplement: Supplementary information [file jexbio-223-222810-s1.pdf]

Supplementary information

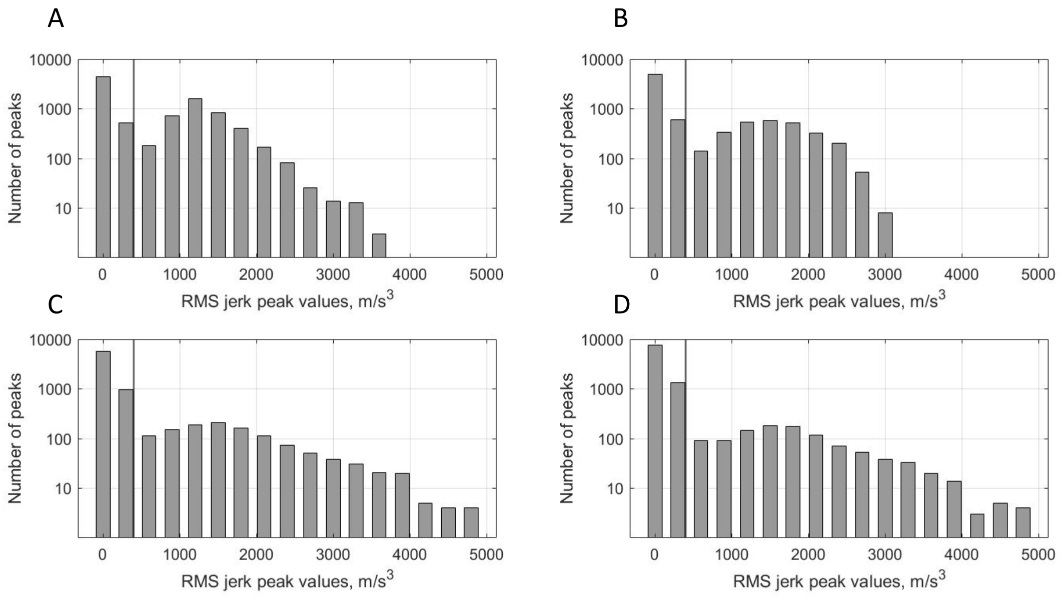

Figure S1: RMS jerk log-histograms for each animal showed a bimodal distribution with a minimum value of about 350 ms^{-3} separating the low and high jerk modes (indicated by the vertical line on each graph). The low jerk mode was associated with swimming and resting behaviours while the higher jerks occurred as brief transients during deep dives and so presumably indicate PCAs. This value was therefore used as a threshold to detect PCAs on all animals. A: KER17, B: KER18, C: PV18_1, D: PV18_2

Journal of Experimental Biology • Supplementary information

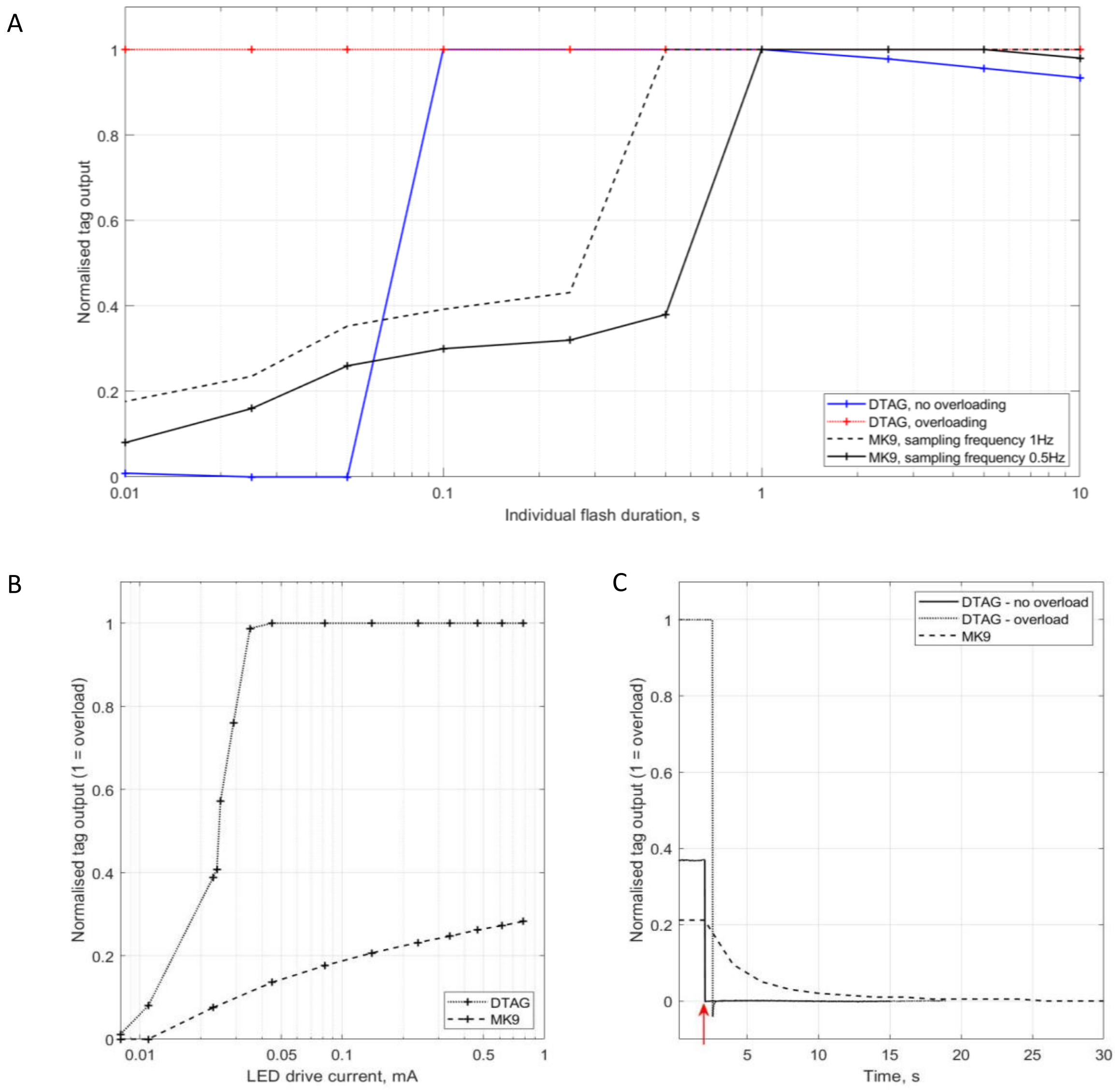

Figure S2: The frequency response, sensitivity and decay time of the light sensors contained in the MK9 and the DTAG-4 were measured to establish their capacity to detect bioluminescent flashes using a 460 nm blue LED at 13.5 cm distance as a light source in an otherwise dark box (approx. ambient light level of 10^{-10} W.cm^{-2}). The MK9 light sensor is intended for ambient light level measurement and so has a lower sampling rate and bandwidth but a wider dynamic range (achieved with a logarithmic response) than the DTAG. The tags were configured in the same way as during field deployments on SES - see Methods in the current study and in Vacquié-Garcia *et al.* (2012, 2017). **A**: Frequency responses of the sensors in terms of responses to light pulses of varying length. The light sensor outputs are

normalised so that 1 corresponds to the received flash intensity at low frequencies and 0 to the noise level (i.e. when there was no flash). Individual flash duration was varied from 0.01 s to 10 s with x s between flashes. LED current was: DTAG (no overload): 0.025 mA, DTAG (overload): 0.8 mA, MK9: 0.3 mA. Sampling frequency: DTAG: 50Hz, MK9: 1 and 0.5 Hz. The shortest flashes reliably detected by the tags were 1 s for the MK9 at 1 Hz sampling rate and 0.1 s for the DTAG. **B**: Comparison of sensor sensitivity using fixed duration flashes with varying intensities. Responses are normalised to clipping level (DTAG: 0.9 arb. Unit, MK9: 250 arb. Unit). The flash duration was kept constant for each tag: DTAG: 2 s, MK9: 4 s. The MK9 responded to all tested flash intensities without overloading while the DTAG overloaded for intensities > 2.8×10^{-9} $W.cm^{-2}$. **C**: Decay times to 1% of initial level for the DTAG (with and without overload) and MK9, synchronised to the end of a 5s duration flash (red arrow). The DTAG decay time to 1% of initial level averaged 40 ms (250 ms when overloaded) compared to 30 s for the MK9.

Journal of Experimental Biology • Supplementary information
